# The role of mitochondria-endoplasmic reticulum crosstalk in colorectal cancer

**DOI:** 10.1016/j.gendis.2025.101766

**Published:** 2025-07-08

**Authors:** Lanshu Xiao, Yao Wei, Yiping Qin, Bianqin Guo

**Affiliations:** a Chongqing University Cancer Hospital, School of Medicine, Chongqing University, Chongqing 400044, China; b Chongqing Key Laboratory of Translational Research for Cancer Metastasis and Individualized Treatment, Chongqing University Cancer Hospital, Chongqing 400030, China

**Keywords:** CRC, ER, MAMs, Mitochondria, Therapeutic strategy

## Abstract

Colorectal cancer (CRC) is a significant health burden globally, with the third highest incidence and the second highest mortality among all types of cancer. Understanding the mechanisms underlying CRC progression is crucial for advancing therapeutic strategies. Organelles are essential components of cells and play a critical role in the initiation and progression of cancer. Over the past decades, numerous studies have demonstrated that mitochondria and the endoplasmic reticulum (ER) can communicate through signaling pathways, thereby regulating cellular homeostasis and function in both normal and cancer cells. This interaction primarily occurs through mitochondria-associated endoplasmic reticulum membranes (MAMs). MAMs, as key nodes in cancer initiation and progression, are also potential vulnerabilities of cancer cells, offering promising opportunities for cancer treatment. Recent research further emphasizes the close association between MAMs and CRC in terms of proliferation, apoptosis, and invasion. To deepen our understanding of the interactions and mechanisms between mitochondria and the ER in CRC, this review, for the first time, synthesizes the research advancements concerning the crosstalk between these organelles in CRC. It innovatively identifies potential targets associated with MAMs, aiming to uncover novel therapeutic strategies for CRC.

## Introduction

According to the latest global cancer statistics report for 2022, colorectal cancer (CRC) ranks third in incidence and second in mortality.^1^ The risk factors associated with CRC include aging populations, male gender, obesity, genetic factors, environmental influences, sedentary lifestyle, smoking, and dietary habits.^2^, ^3^, ^4^ Due to the subtle presentation of CRC in its early stages and the challenges in differential diagnosis, approximately 20% of patients are diagnosed with metastatic CRC at the time of initial diagnosis. Additionally, about 50% of localized CRC cases progress to tumor metastasis as the disease advances, leading to the development of metastatic CRC.^3^^,^^5^ The prognosis for patients with metastatic CRC is unfavorable, with a less than 20% 5-year survival rate.^3^ Consequently, CRC not only imposes a significant societal burden but also represents a substantial threat to human health.

Organelles are vital cellular components that play crucial roles in tumor initiation and progression. Alterations in organelles can serve as indicators for assessing the risk and prognosis of tumors.^6^ Mitochondria are crucial organelles present in nearly all eukaryotic cells. They play central roles in various cellular processes such as ATP production, lipid metabolism, oxidative phosphorylation, Ca^2+^ homeostasis, and the generation of reactive oxygen species (ROS), making them pivotal for cellular metabolism.^7^, ^8^, ^9^ Given their significance in maintaining energy balance, mitochondrial dysfunction can contribute to the development of various diseases, including developmental disorders, neuromuscular conditions, metabolic disorders, and cancer.^10^, ^11^, ^12^, ^13^ The endoplasmic reticulum (ER), the largest organelle in most cells, is involved in functions such as protein and lipid synthesis, Ca^2+^ regulation, and modulation of intracellular signaling pathways.^14^, ^15^, ^16^ ER dysfunction can lead to ER stress and activation of the unfolded protein response (UPR), which is implicated in diseases such as diabetes, neurodegenerative disorders, cardiovascular ailments, and stroke, while also playing a critical role in tumor growth and chemoresistance.^17^^,^^18^

Over the past few decades, extensive research has demonstrated that mitochondria and the ER can communicate through signaling pathways, thereby regulating cellular homeostasis and function in both normal cells and cancer cells. These interactions involve crucial processes such as Ca^2+^ homeostasis regulation, lipid metabolism, mitochondrial dynamics, autophagy, and ER stress.^19^, ^20^, ^21^ In the early 1990s, researchers identified a membrane structure facilitating interaction between mitochondria and the ER, known as the mitochondria-associated endoplasmic reticulum membranes (MAMs).^22^ MAMs serve as a platform for communication between mitochondria and the ER, revealing a close coupling of dynamics and molecular information exchange between these two organelles.^23^ Besides, as key nodes in cancer initiation and progression, MAMs are also potential vulnerabilities of cancer cells, offering promising opportunities for cancer treatment.^24^^,^^25^ Recent research further emphasizes the close association between MAMs and CRC in terms of proliferation, apoptosis, and invasion.^26^, ^27^, ^28^ To deepen our understanding of the interactions and mechanisms between mitochondria and the ER in CRC, this review, for the first time, synthesizes the research advancements concerning the crosstalk between these organelles in CRC. It innovatively identifies potential targets associated with MAMs, aiming to uncover novel therapeutic strategies for CRC.

## Overview of mitochondria, ER, and MAMs, and implications of MAMs in tumorigenesis

### Mitochondria

**Figure 1:**
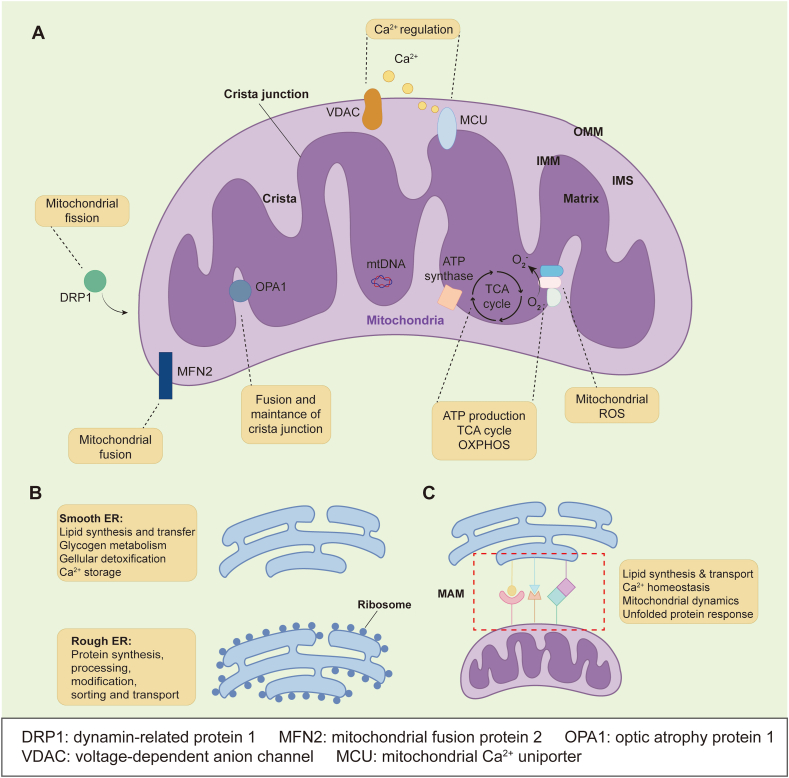
The function of mitochondria, endoplasmic reticulum (ER), and mitochondria-associated ER membranes (MAMs). **(A)** Mitochondria consist of the outer mitochondrial membrane (OMM), inner mitochondrial membrane (IMM), intermembrane space (IMS), and matrix. The OMM contains a number of proteins, such as voltage-dependent anion channel (VDAC), which keeps Ca^2+^ homeostasis in mitochondria, and regulators of mitochondrial fusion, such as mitochondrial fusion protein 2 (MFN2). Mitochondrial fission is mediated by the recruitment of dynamin-related protein 1 (DRP1) on the OMM. Mitochondrial Ca^2+^ uniporter (MCU) is a major conduit in the IMM, mediating Ca^2+^ uptake into the mitochondrial matrix. Mitochondrial cristae are membrane invaginations and host respiratory chain complexes and adenosine triphosphate (ATP) synthase for oxidative phosphorylation (OXPHOS) that produces ATP and mitochondrial reactive oxygen species (ROS) as byproducts. Optic atrophy protein 1 (OPA1) forms oligomers at cristae junctions. The matrix serves as the site of the tricarboxylic acid (TCA) cycle and mitochondrial deoxyribonucleic acid (mtDNA) replication. **(B)** The main structural difference between the smooth ER and the rough ER is that the rough ER is covered with ribosomes. **(C)** MAMs are recognized as a critical platform for the interaction between mitochondria and the ER, with numerous proteins that maintain the structure and function of MAMs playing essential roles.

A complete mitochondrion consists of the outer mitochondrial membrane (OMM), inner mitochondrial membrane (IMM), intermembrane space (IMS), and matrix (Fig. 1A).^29^^,^^30^ The OMM, composed of various lipids and proteins, is a lipid bilayer that surrounds the entire mitochondrion.^31^ The main functions of OMM include serving as a barrier between the mitochondria and the cytoplasm, mediating the transport of metabolites and ions in and out of the mitochondria, and participating in various intracellular signaling pathways.^32^ The IMM is a highly folded membrane and forms cristae, providing a larger surface area and space, which is beneficial to energy metabolism within the mitochondria, such as oxidative phosphorylation, ATP production, Ca^2+^ homeostasis, and ROS generation.^29^^,^^33^, ^34^, ^35^ The IMS within mitochondria, being the smallest and narrowest region, plays a crucial role in numerous cellular processes.^29^^,^^35^ IMS proteins participate in the transport of proteins, lipids, metabolites, and metal ions. They are also fundamental to signal transduction and metabolism, and maintain the ultrastructure of the mitochondria.^35^ The space surrounded by the IMM, known as the mitochondrial matrix, is a liquid region within the mitochondria containing various metabolic products, enzymes, ribosomes, proteins, and mitochondrial DNA. Its unique structure and composition provide a favorable site for different biochemical reactions such as protein biosynthesis, lipid biosynthesis, the Krebs cycle, oxidative phosphorylation, and mitochondrial DNA replication.^29^

### Endoplasmic reticulum

The ER is the largest organelle in most cells, characterized by a complex structure comprising the nuclear envelope and the peripheral ER. The peripheral ER extends from the nuclear envelope into the cytoplasm, forming a network of sheets and dynamic tubules.^36^ The sheets of the ER are studded with ribosomes, known as rough ER, which play a key role in protein synthesis, processing, modification, sorting, and transport. The dynamic tubules of the ER, which have fewer ribosomes on their surface, are commonly referred to as smooth ER. They are involved in lipid transfer and contact with other organelles like mitochondria. In addition, they participate in lipid synthesis, glycogen metabolism, and cellular detoxification, and serve as sites for Ca^2+^ storage (Fig. 1B).^37^^,^^38^

### Mitochondria-associated ER membranes

The interaction and contact between organelles are increasingly recognized as pivotal for controlling cellular behavior. Given the extensive network of the ER within the cytoplasm, establishing connections with other organelles appears to be a straightforward process for the ER. The physical interaction sites between the ER and OMM were first observed by Bernhard et al in 1952 using electron microscopy.^39^ It was not until 1994 that the membrane structure between the ER and mitochondria was named MAM.^40^ While structurally comprised of the OMM and ER, MAMs depend not only on the close physical proximity of these membranes but also on the orchestrated regulation of specific proteins. Recent investigations have unveiled a plethora of proteins that play crucial roles in preserving the structure and functionality of MAMs.^41^, ^42^, ^43^, ^44^ Due to the distinctive structural attributes of MAMs, they exhibit functional characteristics of both organelles, including participating in lipid synthesis and transport, and maintenance of Ca^2+^ homeostasis.^25^ Moreover, MAMs are actively involved in governing ER stress responses, mitochondrial dynamics such as fission/fusion, and the regulation of autophagy processes (Fig. 1C).^45^^,^^46^

### Implications of MAMs in tumors

Numerous studies have demonstrated that MAMs influence tumors mainly by regulating Ca^2+^ homeostasis, lipid metabolism, mitochondria morphology, and ER stress. Regulation of Ca^2+^ signaling is critical in cancer as it is involved in epithelial-to-mesenchymal transition, invasion, and resistance to apoptosis.^47^ Therefore, Ca^2+^-associated proteins in MAMs play crucial roles in cancer development. The inositol 1,4,5-trisphosphate receptor (IP3R)-glucose-regulated protein 75 (GRP75)-voltage-dependent anion channel (VDAC)-mitochondrial Ca^2+^ uniporter (MCU) signaling axis is important in Ca^2+^ transport and is regulated by proteins such as phosphatase and tensin homolog deleted on chromosome ten (PTEN), breast cancer type 1 susceptibility protein (BRCA1), and B-cell lymphoma-2 (BCL-2), which can affect apoptosis of tumor cells.^20^^,^^48^ Elevated lipid levels in cancer cells promote proliferation and act as energy stores and messengers in carcinogenic pathways.^49^ Notably, the expression of multiple lipid-related enzymes is upregulated in various cancers, including lung, ovarian, and prostate cancer.^50^^,^^51^ Moreover, key enzymes involved in lipid synthesis, such as fatty acid CoA ligase (FACL/ACSL) and acyl-CoA: cholesterol acyltransferase 1 (ACAT1/SOAT1), are mainly located in MAMs.^40^^,^^52^ The changes of mitochondrial morphology have been observed in various cancer cells such as glioblastoma, lung cancer, and metastatic breast cancer.^53^ Mitochondrial morphology is mainly regulated by mitochondrial fusion protein 2 (MFN2) and dynamin-related protein 1 (DRP1).^54^ It is worth noting that these proteins are crucial for connecting the ER to mitochondria and stabilizing MAMs.^54^ Studies have shown that inhibiting DRP1 or overexpressing MFN2 to restore fusion phenotypes can impair cell growth and increase cell apoptosis, indicating the important role of mitochondrial dynamics in tumor development.^19^ In cancer cells, ER signaling pathways are frequently dysregulated to promote metabolism. UPR plays an important role in cancer cell growth, metastasis, and survival.^55^ A close interplay exists between the components of the MAMs and the UPR, with several ER chaperone proteins like immunoglobulin heavy chain binding protein (BiP), protein kinase R-like endoplasmic reticulum kinase (PERK), and MFN2 being present in the MAMs.^42^^,^^56^^,^^57^ Remarkably, MAMs play a crucial role in promoting cancer cell proliferation and cell death, providing a platform for regulating ER stress.^42^

MAMs are also recognized as pivotal hubs in the regulation of cell apoptosis and tumor growth by providing a shelter for both anti-cancer and pro-cancer proteins. Among the extensively studied anti-cancer proteins, p53 stands out as a key regulator of tumorigenesis through a Ca^2+^-dependent pathway.^58^ Notably, p53 has been identified to localize within MAMs.^59^ Its function involves promoting the accumulation of Ca^2+^ in mitochondria, leading to alterations in organelle morphology and the initiation of cell apoptosis.^59^ Another tumor suppressor protein within MAMs, Brca1-associated protein 1 (BAP1), facilitates Ca^2+^ influx into mitochondria by interacting with IP3R.^60^ Mutations in BAP1 have been linked to various cancers, including renal cell carcinoma and melanomas, where aberrant function can disrupt Ca^2+^ homeostasis in mitochondria, impacting apoptosis regulation and contributing to carcinogenesis.^61^ Additionally, the oncogene H-RAS exerts its anti-apoptotic function by modulating Ca^2+^-dependent apoptosis.^62^ Furthermore, caveolin-1 (CAV1), a membrane protein associated with carcinogenesis and located on MAMs, plays a role in cholesterol efflux.^63^ Its up-regulation has been observed in diverse cancers and is implicated in cancer progression, metastasis, and drug resistance.^64^, ^65^, ^66^ Proto-oncogene protein BCL-2, situated in MAMs, and MAM-regulated protein sigma-1 receptor (Sig-1R), modulated by ER stress, exhibit anti-apoptotic effects, with Sig-1R influencing cellular survival through the regulation of BCL-2 expression.^67^, ^68^, ^69^ Therefore, studying the specific proteins of MAMs is of great significance for cancer treatment.

## ER-mitochondria crosstalk in CRC

The intricate communication between mitochondria and the ER has emerged as a critical determinant of cellular homeostasis and functionality.^70^ Disruption of this crosstalk has been implicated as a key factor in the pathogenesis of various diseases.^71^ Fundamental intracellular pathways, including Ca^2+^ signaling, lipid metabolism, mitochondrial dynamics, autophagy, and ER stress, underscore the significance of the crosstalk between the ER and mitochondria.^72^, ^73^, ^74^ Particularly, investigations into the molecular mechanisms underlying tumorigenesis increasingly focus on the context of ER-mitochondria communication. The role of MAMs has garnered significant attention and serves as a focal point of research endeavors.^24^ This review aims to elucidate the regulatory influence of MAMs on the behavior of cancer cells in CRC, providing insights into their potential implications in disease progression and therapeutic interventions.

## Ca^2+^ transfer and CRC

### Ca^2+^ transfer at the ER-mitochondria interface

**Figure 2:**
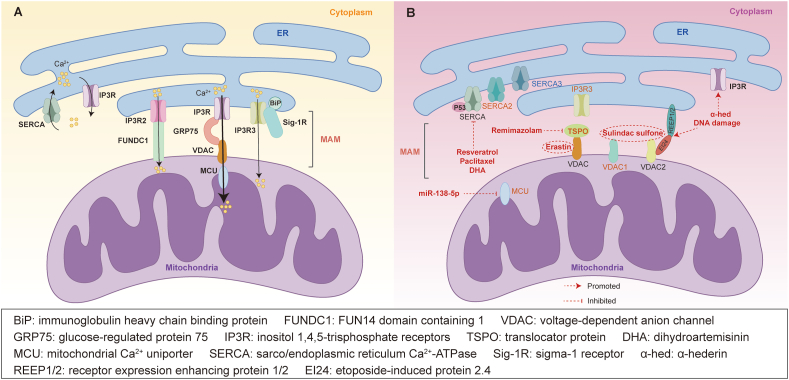
Ca^2+^ transfer in endoplasmic reticulum (ER), mitochondria, and mitochondria-associated ER membranes (MAMs). **(A)** Ca^2+^ transfer in physiology. Sarco/endoplasmic reticulum Ca^2+^-ATPase (SERCA) is responsible for transporting Ca^2+^ from the cytoplasm into the ER. The inositol 1,4,5-trisphosphate receptor (IP3R)-glucose-regulated protein 75 (GRP75)-voltage-dependent anion channel (VDAC) complex helps maintain MAM structure and facilitates Ca^2+^ uptake by the outer mitochondrial membrane (OMM). Sigma-1 receptor (Sig-1R) forms a Ca^2+^-sensitive complex with immunoglobulin heavy chain binding protein (BiP), aiding in the transmission of Ca^2+^ signals from the ER to the mitochondria by stabilizing IP3R3. FUN14 domain containing 1 (FUNDC1) interacts with IP3R2 to form a protein bridge connecting the ER and mitochondria, participating in Ca^2+^ transfer from the ER to the mitochondria and the formation of MAM. Ca^2+^ can only enter the inner mitochondrial membrane (IMM) through mitochondrial Ca^2+^ uniporter (MCU) with lower Ca^2+^ affinity. **(B)** The Ca^2+^ homeostasis and therapy targets in colorectal cancer (CRC). During the development of CRC, SERCA2 expression significantly increases while SERCA3 expression decreases. Resveratrol, paclitaxel, and dihydroartemisinin (DHA) can reduce SERCA activity. In CRC cells undergoing anti-cancer treatment, p53 accumulates and interacts directly with the SERCA pump to regulate Ca^2+^ homeostasis and enhance Ca^2+^ transport to the mitochondria. α-hederin induces paraptosis in CRC by increasing the expression of IP3R and activating the Ca^2+^ signaling pathway. Overexpression of IP3R3 inhibits apoptosis in CRC cells. Remimazolam targets translocator protein (TSPO) to inhibit the TSPO/VDAC pathway, leading to increased apoptosis in CRC cells. Erastin induces apoptosis by binding to VDAC. Etoposide-induced protein 2.4 (EI24) interacts with VDAC2, enhancing Ca^2+^ flux from the ER to mitochondria, promoting DNA damage-induced apoptosis. VDAC1 is overexpressed in CRC. Sulindac sulfone induces apoptosis in human CRC cells by binding to VDAC1/2. MCU protein levels are significantly increased in CRC. The inhibitory effect of miR-138-5p on MCU is highlighted in CRC. The orange figure indicates overexpression. The blue figure indicates underexpression.

Ca^2+^ signals typically originate from the ER and lysosomes, influencing other organelles through the creation of microdomains at membrane contact sites.^75^^,^^76^ Studies have demonstrated that Ca^2+^ signals released from the ER primarily target mitochondria, contributing to the maintenance of intracellular Ca^2+^ homeostasis.^77^ During this process, numerous ER, mitochondria, and MAM-resident proteins mediate Ca^2+^ transport through interaction between the ER and mitochondria (Fig. 2A).^78^^,^^79^

The ER functions as the primary intracellular Ca^2+^ storage organelle.^17^^,^^80^ Two crucial ER-resident proteins involved in Ca^2+^ transport and highly concentrated in the MAMs are the sarco/endoplasmic reticulum Ca^2+^-ATPase (SERCA) and IP3R. SERCA, encoded by three genes (SERCA1, SERCA2, and SERCA3), is predominantly located on the ER membrane and MAMs.^81^^,^^82^ SERCA is responsible for transporting Ca^2+^ from the cytoplasm into the ER, establishing a Ca^2+^ gradient between the cytoplasm and the ER to regulate Ca^2+^ levels within the ER.^83^ IP3R, situated on the ER surface, acts as a crucial Ca^2+^ efflux channel and plays a key role in Ca^2+^ signaling to the mitochondria within the MAMs.^84^ Additionally, the ER protein Sig-1R, localized in MAMs, serves as a marker for MAMs.^85^ Sig-1R forms a Ca^2+^-sensitive complex with BiP, aiding in the transmission of Ca^2+^ signals from the ER to the mitochondria by stabilizing type 3 IP3R (IP3R3).^86^ OMM proteins, such as VDAC and GRP75, help maintain MAM structure and facilitate Ca^2+^ uptake by the OMM.^77^ VDAC, an ion channel on the OMM, has three isoforms in mammalian cells (VDAC1, VDAC2, and VDAC3).^87^ The VDAC1 and VDAC2 complex with IP3R-GRP75 is crucial for Ca^2+^ uptake by mitochondria.^88^^,^^89^ In addition to the IP3R-GRP75-VDAC complex, another OMM protein, FUN14 domain containing 1 (FUNDC1), interacts with type 2 IP3R (IP3R2) to form a protein bridge connecting the ER and mitochondria, participating in Ca^2+^ transfer from the ER to the mitochondria and the formation of MAMs.^90^ For Ca^2+^ uptake by the IMM, the MCU is crucial to the process. Unlike VDAC, which allows free Ca^2+^ passage through the OMM, Ca^2+^ can only enter the IMM through MCU with a lower Ca^2+^ affinity.^91^ MCU tightly regulates Ca^2+^ concentration in the mitochondrial matrix, crucial for maintaining normal cellular function.^92^

### Roles of ER-mitochondria Ca^2+^ signaling in CRC

The exchange of Ca^2+^ between the ER and mitochondria has a significant impact on triggering apoptosis in cancer cells. Mitochondria rely on adequate Ca^2+^ uptake to sustain enzyme activity and viability, while cancer cells are highly sensitive to disruptions in Ca^2+^ transfer to the mitochondria.^93^^,^^94^ Oncogenes or tumor suppressors can perturb Ca^2+^ homeostasis by interacting with MAM-resident proteins, thereby directly modulating the flow of Ca^2+^ from the ER to the mitochondria, influencing the fate of cancer cells.^95^ This section outlines the dysregulation of Ca^2+^ homeostasis centered on ER-mitochondrial interactions in CRC, and discusses targeted therapeutic approaches aimed at restoring Ca^2+^ balance (Fig. 2B).

Recent investigations have revealed alterations in the expression levels of various Ca^2+^ signaling proteins on MAMs in CRC, including SERCA, IP3R, VDAC, and MCU.^24^ As previously mentioned, SERCA is encoded by three genes, with SERCA2 and SERCA3 being the focus of CRC research. Studies have indicated that in CRC, SERCA2 expression is notably elevated compared with adjacent normal tissues.^96^ Overexpression of SERCA2 can stimulate the proliferation and migration of SW-480 cells by activating the mitogen-activated protein kinase (MAPK) and protein kinase B (Akt) signaling pathways, making it a pivotal molecular determinant in CRC onset and progression.^97^ On the other hand, during CRC development, decreased SERCA3 expression leads to progressive disturbances in cellular Ca^2+^ homeostasis, considered an early event in colon carcinogenesis.^98^^,^^99^ Recent research has demonstrated that resveratrol and its derivative paclitaxel inhibit ATP synthase, subsequently diminishing SERCA activity in MAMs, resulting in cancer cell death. Resveratrol also impacts ER-mitochondria contact, inducing mitochondrial Ca^2+^ overload and promoting cancer cell death.^100^ The anti-cancer efficacy of resveratrol is currently under exploration in clinical trials for CRC patients.^19^^,^^42^^,^^101^ Moreover, dihydroartemisinin, another anti-cancer compound studied in CRC, has been shown to induce ER stress by inhibiting SERCA activity, leading to Ca^2+^ overload and apoptosis in HCT-116 cells.^102^ Giorgi et al observed that in CRC HCT-116 cells undergoing anti-cancer treatment, the tumor suppressor gene p53 accumulates in the ER and MAMs, modulating Ca^2+^ homeostasis. Specifically, p53 interacts directly with the SERCA pump, altering the ER’s oxidative state, increasing Ca^2+^ load, and enhancing its transfer to the mitochondria. Conversely, pharmacological suppression of p53 inhibits SERCA pump activity, reducing Ca^2+^ signaling from the ER to the mitochondria.^58^

Studies have demonstrated that IP3R is situated on the surface of the ER and is crucial for Ca^2+^ homeostasis in CRC cells.^103^^,^^104^ Therefore, targeting IP3R to disrupt Ca^2+^ homeostasis in CRC cells is one of the strategies for the treatment of CRC. For example, α-hederin, a natural plant agent, can induce paraptosis by increasing the expression of IP3R and activating the Ca^2+^ signaling pathway in CRC. Additionally, α-hederin induces paraptosis in 5-fluorouracil-resistant CRC cells, and it reduces the growth of 5-fluorouracil-resistant CRC xenografts.^105^ Currently, three IP3R subtypes (IP3R1/2/3) are identified. Interestingly, compared with IP3R1 and IP3R2, IP3R3 has a different function in tumor cells.^106^^,^^107^ Previous investigations have assessed the expression of these IP3R subtypes in 116 surgically excised CRC tissues. IP3R1 and IP3R2 were found to be expressed in both non-cancerous and CRC tissues, whereas IP3R3 expression was exclusive to CRC tissues. Notably, the expression levels of IP3R3 directly correlated with the invasiveness of CRC. Studies have demonstrated that knockdown of IP3R3 promotes apoptosis, while its overexpression inhibits apoptosis in CRC cell lines.^108^

Furthermore, OMM protein VDAC and IMM protein MCU play essential roles in CRC proliferation and invasion. Translocator protein (TSPO), a peripheral benzodiazepine receptor, interacts with VDAC and affects its expression. Liu et al reported that TSPO was highly expressed in CRC, and targeting TSPO with remimazolam inhibited the TSPO/VDAC pathway, resulting in increased apoptosis in CRC cells.^109^ Recent studies have shown a notable expansion of the ER in response to DNA damage in CRC HCT-116 cells. This process relies on the activation of ER, forming receptor expression enhancing protein 1 (REEP1), REEP2, and etoposide-induced protein 2.4 (EI24), mediated by p53. Subsequently, EI24 interacts with VDAC2, promoting the formation of ER-mitochondria contacts, enhancing Ca^2+^ flux from the ER to the mitochondria, and promoting DNA damage-induced apoptosis.^110^ Moreover, VDAC1 is overexpressed in CRC, and its down-regulation can hinder the proliferation of CRC cell lines.^111^^,^^112^ Erastin, a newly discovered small molecule, binds to VDAC and disrupts normal mitochondrial function, leading to the opening of the mitochondrial permeability transition pore and caspase-9-dependent cell apoptosis in CRC cells HT-29.^113^ Sulindac sulfone, another compound, induces apoptosis in human colon cancer cells by binding to VDAC1/2 and is considered a promising chemopreventive drug with minimal side effects for colon cancer.^114^^,^^115^ The role of MCU in CRC has attracted attention due to its involvement in regulating Ca^2+^ accumulation in the mitochondrial matrix. Studies have shown that MCU mRNA and protein levels are significantly elevated in CRC tissues and are correlated with poor prognosis in CRC patients. Up-regulation of MCU enhances mitochondrial Ca^2+^ uptake, promoting mitochondrial biogenesis and accelerating CRC development.^116^ Zhu et al have identified a miRNA molecule that targets MCU, highlighting the inhibitory effect of miR-138-5p on MCU. Their results indicate that miR-138-5p leads to decreased expression of MCU, which subsequently reduces the level of mitochondrial Ca^2+^ to inhibit CRC growth.^117^

## Lipid metabolism and CRC

### Lipid synthesis and metabolism at the ER-mitochondria interface

The ER acts as the primary site for lipid synthesis, such as phosphatidylserine (PS) and phosphatidylcholine (PC), while the enzymes responsible for synthesizing these lipids are mainly located in the mitochondria, ER, and MAMs.^40^^,^^118^ The process involves PS synthase 1/2 (PSS1/2) localized in MAMs synthesizing PS in the ER, which is then transported to mitochondria, aided by ATP. Within the mitochondria, PS is converted to phosphatidylethanolamine (PE) by the phosphatidylserine decarboxylase (PSD) in the IMM. PE is subsequently exported back to the ER, where PE N-methyltransferase 2 (PEMT2) further modifies it into PC.^55^^,^^119^ PEMT2 is also localized on MAMs.^120^

In general, lipids are transported between specific organelles through vesicles. However, lipids cannot enter mitochondria through vesicles even when they are needed.^121^ In this case, several MAM proteins regulate the non-vesicular transport of lipids from the ER to mitochondria.^25^ Lipid transfer proteins like oxysterol binding related proteins 5/8 (ORP5/8), enriched in MAMs, facilitate the transfer of PS from the ER to mitochondria by interacting with protein tyrosine phosphatase interacting protein 51 (PTPIP51).^122^ Tp53-regulated inhibitor of apoptosis 1 (TRIAP1) interacts with protein of relevant evolutionary and lymphoid interest (PRELI) family proteins to facilitate intramitochondrial lipid transfer for PE synthesis.^123^^,^^124^ This intricate lipid transport and synthesis network is vital for cellular function and offers potential therapeutic targets for lipid-related diseases.

MAMs also play a significant role in cholesterol metabolism, housing key enzymes like long-chain acyl-CoA synthetase 4 (ACSL4/FACL4), ACAT1, steroidogenic acute regulatory protein (StAR), and ATPase family AAA domain-containing protein 3 (ATAD3).^125^^,^^126^ In the basal or resting state, ACAT1 in the MAMs catalyzes the formation of cholesterol ester from free cholesterol to maintain the balance between membrane-bound cholesterol and cytoplasmic lipid droplet-stored cholesterol. Under acute stress or hormonal stimulation, MAM-associated StAR induces cholesterol entry into mitochondria by interacting crucially with VDAC2, thereby initiating mitochondrial steroidogenesis.^127^ Additionally, ATAD3 is enriched in the MAMs and is believed to regulate steroidogenesis by participating in MAMs formation and transferring cholesterol between the ER and mitochondria.^126^

**Figure 3:**
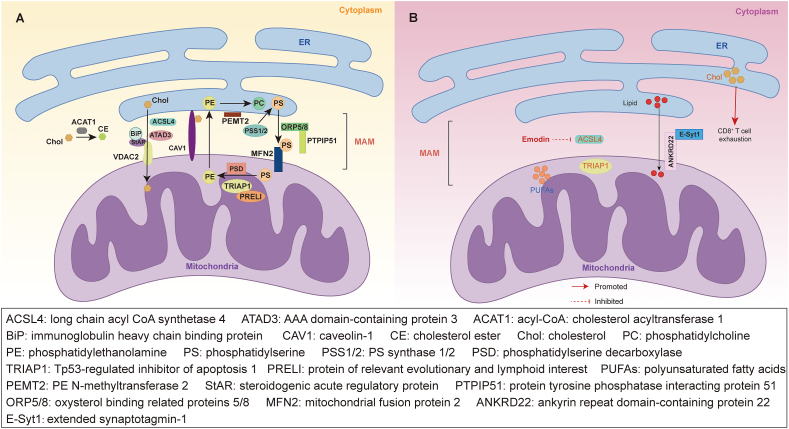
Lipid synthesis and metabolism in endoplasmic reticulum (ER), mitochondria, and mitochondria-associated ER membranes (MAMs). **(A)** Lipid synthesis in physiology. Phosphatidylserine (PS) synthase 1/2 (PSS1/2) synthesize PS in the ER. Oxysterol binding related protein 5/8 (ORP5/8) interacts with protein tyrosine phosphatase interacting protein 51 (PTPIP51) to promote the transfer of PS from the ER to mitochondria. PS is converted to phosphatidylethanolamine (PE) through phosphatidylserine decarboxylase (PSD) in the inner mitochondrial membrane (IMM). TRIAP1 interacts with PRELI to facilitate lipid transfer within mitochondria for PE synthesis. Mitochondrial fusion protein 2 (MFN2) binds to PS in MAMs, promoting PE synthesis. PEMT2 further modifies PE to PC in the ER. Long-chain acyl-CoA synthetase 4 (ACSL4) plays a significant role in cholesterol metabolism. ACAT1 catalyzes the generation of CE from free cholesterol. Immunoglobulin heavy chain binding protein (BiP) properly folds StAR, enabling StAR to interact with voltage-dependent anion channel 2 (VDAC2) under acute stress, inducing cholesterol entry into mitochondria. ATAD3 is implicated in mediating the formation of MAMs and facilitating the transfer of cholesterol between the ER and mitochondria. CAV1 contributes to the accumulation of free cholesterol in MAMs and stabilization of ER-mitochondria contact sites. **(B)** Reprogramming of lipid metabolism and therapy targets in colorectal cancer (CRC). ANKRD22 collaborates with extended synaptotagmin-1 (E-Syt1) to stimulate lipid transport to mitochondria. The level of polyunsaturated fatty acids (PUFAs) is downregulated in CRC. Tp53-regulated inhibitor of apoptosis 1 (TRIAP1) and long-chain acyl CoA synthetase 4 (ACSL4) are overexpressed in CRC. Emodin inhibits ACSL4. Elevated cholesterol levels in CRC can trigger CD8^+^ T cell exhaustion. The orange figure indicates overexpression. The blue figure indicates underexpression.

Due to the complex structure and heterogeneity of MAMs, they require specific protein and lipid components to maintain the formation of the interface. Cholesterol, a critical component for MAMs stability, influences the interaction between the ER and mitochondria.^128^ CAV1 in MAMs is crucial for the accumulation of free cholesterol within MAMs and the stability of ER-mitochondria contact sites.^129^ ER chaperone BiP in MAMs aids in proper StAR folding for cholesterol synthesis and transport.^130^ MFN2 on MAMs is instrumental in bridging ER-mitochondria interactions, maintaining mitochondrial metabolism and energy balance.^131^ Depletion of MFN2 affects lipid biosynthesis, as it binds to PS in MAMs, promoting PE synthesis.^132^ In conclusion, lipid synthesis and metabolism are essential functions of MAMs, critical for overall cellular health (Fig. 3A).^133^^,^^134^

### Roles of lipid synthesis and metabolism between the ER and mitochondria in CRC

Elevated lipid levels promote tumor cell proliferation and act as an energy store and messenger in carcinogenic pathways.^49^ Reprogramming lipid metabolism can significantly impact various biological processes in CRC, including proliferation, migration, invasion, apoptosis, and other behaviors.^135^ The intricate crosstalk between mitochondria and the ER is pivotal for lipid transport and synthesis in CRC (Fig. 3B). Here, we outline the implications of dysregulated mitochondrial-ER lipid metabolism in CRC.

Recent studies have shed light on the association between lipid transport proteins and the development of CRC. Ankyrin repeat domain-containing protein 22 (ANKRD22), a nuclear-encoded mitochondrial membrane protein, is activated by the tumor microenvironment and up-regulated in CRC stem cells. ANKRD22 promotes glycolysis and collaborates with the lipid transfer protein extended synaptotagmin 1 (E-Syt1) to stimulate lipid transport to the mitochondria, leading to a reduction in mitochondrial quantity. This process drives metabolic reprogramming in CRC cells to meet their metabolic demands.^136^ Increased consumption of polyunsaturated fatty acids has shown an inverse correlation with CRC incidence. Research by Zhang et al has revealed that polyunsaturated fatty acids induce apoptosis in colon cancer cells through a mitochondria-dependent pathway, characterized by disruptions in mitochondrial membrane potential, ROS generation, intracellular Ca^2+^ accumulation, activation of caspase-9 and caspase-3, decreased ATP levels, and an altered Bax/Bcl2 expression ratio.^137^

Furthermore, studies have highlighted the involvement of lipid synthetases in CRC. Nedara et al have demonstrated that the expression of TRIAP1 supports cancer cell proliferation and tumorigenesis in CRC cell models.^28^ Loss of TRIAP1 disrupts mitochondrial ultrastructure without significantly impacting cardiolipin levels and mitochondrial activity.^28^ Depletion of TRIAP1 induces external perturbations in mitochondria, leading to ER-mediated alterations in lipid homeostasis and triggering a p53-mediated stress response.^28^ ACSL4, a peripheral membrane protein predominantly found in MAMs, is pivotal in lipid regulation and steroid synthesis, particularly in the early stages of steroid synthesis.^138^ ACSL4 is up-regulated in colon adenocarcinoma tissues and associated with a poor prognosis in colon adenocarcinoma patients.^139^ Down-regulation of ACSL4 can attenuate cell proliferation and invasion.^27^ Emodin, a natural anthraquinone derivative with immunomodulatory, anti-inflammatory, cytotoxic, and growth inhibitory properties on various tumor cells,^27^^,^^140^ has been shown to bind directly to the ACSL4 target, inhibiting ACSL4 in HCT-116 cells and reducing cell proliferation and invasion *in vitro*.^27^

The alterations in lipid composition within the ER and mitochondria of tumor cells, particularly the increase in cholesterol and phospholipid levels, have been extensively documented in various studies. These changes may influence mitochondrial bioenergetics, thereby regulating the survival of tumor cells and impacting their response to chemotherapy.^141^, ^142^, ^143^, ^144^ Tumor cells demonstrate a heightened affinity for cholesterol, which facilitates their proliferation and growth.^145^ Notably, clinical investigations have revealed a positive association between hypercholesterolemia and the susceptibility to CRC, accompanied by the suppression of cellular immunity in CRC patients. Research conducted by Shuwen et al suggested that elevated cholesterol levels in CRC can trigger ER stress in CD8^+^ T cells, leading to impaired ER-mitochondrial contact site function. This disruption culminates in mitochondrial autophagy, dysregulated mitochondrial energy metabolism, and subsequent exhaustion of CD8^+^ T cells.^146^ In conclusion, high levels of serum total cholesterol increase CRC risk, but this risk may be reduced by high dietary intake of polyunsaturated fatty acids.^147^

## Membrane dynamics and CRC

### Mitochondrial fission/fusion and autophagy at the ER-mitochondria interface

The dynamic nature of the ER and mitochondria leads to changes in the MAMs in response to various physiological and pathological stimuli, playing a pivotal role in regulating mitochondrial morphology and dynamics.^72^ Proteins involved in regulating mitochondrial shape, the balance between fission and fusion, and mitochondrial movement, such as DRP1, mitofusin 1/2 (MFN1/2), and mitochondrial Rho-GTPases, are localized in the MAMs.^131^^,^^148^ Mitochondrial division occurs at the MAMs, where ER tubules physically encircle a segment of the mitochondrial network and recruit DRP1 to induce local constriction on the OMM.^149^ Specifically, DRP1 and other adaptor proteins such as mitochondrial dynamics proteins of 49/51 kDa (MiD49/51), mitochondrial fission factor (MFF), and mitochondrial fission protein 1 (FIS1) are recruited to the MAMs, wrapping around the scission site, leading to constriction of mitochondrial tubules and subsequent mitochondrial division through a GTP-dependent mechanism.^150^^,^^151^ Additionally, Adachi et al demonstrated that DRP1 could shape ER tubules in a GTP-independent manner, enhancing ER-mitochondrial association and facilitating mitochondrial division.^152^ These findings underscore the critical role of DRP1 in mitochondrial fission and ER-mitochondria tethering. In mammals, mitochondrial fusion includes several steps, starting with the activation of dynein-associated GTPases, including MFN1/2 on OMM and mitoguardin and optic atrophy protein 1 (OPA1) on IMM, followed by MFN2 facilitating OMM fusion with GTP hydrolysis, subsequently OPA1 mediating IMM fusion, and finally the mixing of intra-mitochondrial components.^153^^,^^154^ MFN1/2 also play a crucial role in linking mitochondria to the ER. MFN2, located in mitochondria, can form homo-complexes. Additionally, MFN2, located in the ER, can interact with MFN1, located in mitochondria, to form hetero-complexes. Both MFN2/MFN2 homo-complexes and MFN1/MFN2 hetero-complexes can tether the ER and mitochondria to promote the MAMs formation.^131^^,^^155^

Autophagy is a lysosome-mediated intracellular circulatory system that not only has a substantial effect on maintaining cellular homeostasis and integrity but also serves as an alternative source of energy for cells during nutrient-deficient conditions. Besides its influence on mitochondrial fission/fusion, MAMs have been identified as significant sites for autophagosome formation in mammalian cells.^74^ Three-dimensional electron tomography studies have demonstrated the recruitment of key autophagy-related proteins, such as autophagy-related protein 5/14 (ATG5/14), to the MAMs.^74^ Additionally, findings from Wu et al revealed an interaction between FUNDC1 and DRP1 during mitophagy, leading to the recruitment of DRP1 to MAMs, thereby promoting mitochondrial fission and mitophagy.^156^ Emerging evidence suggests that MAM-associated proteins, including MFN2, IP3R, hexokinase 2 (HK2), and phosphofurin acidic cluster sorting protein 2 (PACS2), can interact with autophagy-regulating proteins, facilitating the selective removal of defective organelle components and recycling of major membrane structures.^157^ Moreover, certain MAM-resident and tethering proteins have been reported to regulate autophagy. For instance, the overexpression or down-regulation of vesicle-associated membrane protein-associated protein B (VAPB) and protein tyrosine phosphatase interacting protein 51 (PTPI51) can respectively inhibit or enhance autophagy by modulating the tethering, with these effects on autophagy regulation being associated with the mediation of Ca^2+^ transfer from the ER to the mitochondria.^43^

**Figure 4:**
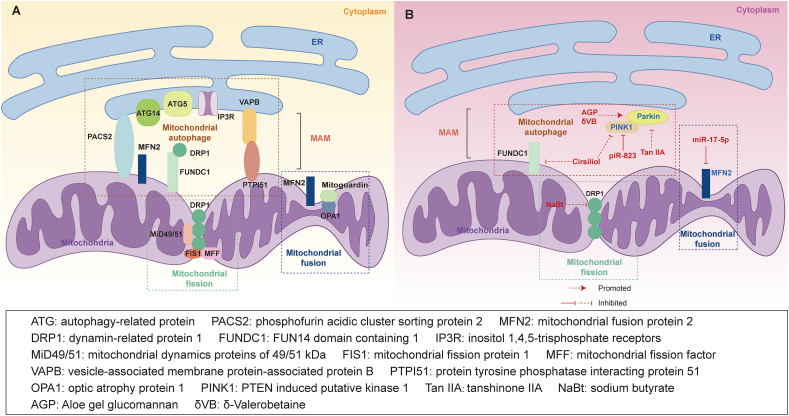
Mitochondrial fusion/fission and autophagy in endoplasmic reticulum (ER), mitochondria, and mitochondria-associated ER membranes (MAMs). **(A)** Mitochondrial fusion/fission and autophagy in physiology. Dynamin-related protein 1 (DRP1), mitochondrial dynamics proteins of 49/51 kDa (MiD49/51), mitochondrial fission factor (MFF), and mitochondrial fission protein 1 (FIS1) encircle the scission sites, leading to mitochondrial fission. Mitochondrial fusion protein 2 (MFN2), mitoguardin, and optic atrophy protein 1 (OPA1) participate in mitochondrial fusion. Autophagy-related protein 14 (ATG14) and ATG5 recruit to the MAMs during mitochondrial autophagy. The interaction between FUN14 domain-containing 1 (FUNDC1) and DRP1 promotes mitochondrial division and mitophagy. MFN2, inositol 1,4,5-trisphosphate receptor (IP3R), and phosphofurin acidic cluster sorting protein 2 (PACS2) can interact with autophagy regulatory proteins to promote membrane structure recycling. Vesicle-associated membrane protein-associated protein B (VAPB)-protein tyrosine phosphatase interacting protein 51 (PTPI51) regulates mitochondrial autophagy by modulating the tethering. **(B)** Mitochondrial fusion/fission and mitophagy and therapy targets in colorectal cancer (CRC). Sodium butyrate (NaBt) down-regulates the expression of DRP1 and inhibits the progression of CRC. Down-regulation of MFN2 by miR-17-5p impairs mitochondrial fusion, ultimately leading to 5-fluorouracil resistance in CRC cells. In human CRC tissues, PTEN-induced putative kinase 1 (PINK1)/Parkin expression is down-regulated. δ-valerobetaine (δVB) and aloe gel glucomannan (AGP) induce mitochondrial apoptosis through the PINK1/Parkin mitophagy pathway. Tanshinone IIA (Tan IIA) promotes apoptosis in CRC cells by inhibiting Parkin-mediated mitochondrial autophagy. Cirsiliol inhibits CRC cell proliferation by disrupting mitochondrial morphology and reducing levels of mitophagy-related proteins, including PINK1, Parkin, and FUNDC1. The non-coding RNA piR-823 interacts with PINK1 to inhibit mitochondrial autophagy and cell apoptosis in CRC. The orange figure indicates overexpression. The blue figure indicates underexpression.

In summary, the interplay between mitochondrial fission/fusion, autophagy, and MAMs underscores the pivotal role of MAMs in determining cell fate (Fig. 4A).^25^^,^^74^

### Roles of mitochondrial fission/fusion and autophagy in CRC

A substantial body of research has highlighted the essential roles of mitochondrial dynamics in cancer progression, sparking a growing interest in the modulation of these mechanisms by MAM-resident proteins.^24^^,^^158^ In this section, our primary focus is on the MAM-mediated regulation of mitochondrial fission, fusion, and mitophagy in the context of CRC progression, along with therapeutic approaches (Fig. 4B).

On the one hand, several studies have suggested that proteins localized in MAMs may impact CRC progression by regulating mitochondrial fission/fusion processes. Excessive mitochondrial fission can trigger cell apoptosis, resulting in damage to cell migration and invasion. As mentioned earlier, mitochondrial fission takes place at the ER-mitochondrial contact site.^149^ DRP1, a key regulator of mammalian mitochondrial fission, co-regulates mitochondrial fission with adaptor proteins situated on MAMs.^159^ Silencing DRP1 via siRNA promotes the accumulation of elongated mitochondria in human CRC HCT-116 and SW-480 cells, leading to reduced proliferation and increased apoptosis. Further exploration has revealed that DRP1 deficiency primarily inhibits cell proliferation and enhances apoptosis in human colon cancer cells by increasing cytochrome C release and reducing mitochondrial membrane potential.^160^ MFN2, a crucial player in mitochondrial fusion, is down-regulated in CRC tissues. Down-regulation of MFN2 signifies a poor prognosis and acts as an independent risk factor for colon cancer prognosis.^161^

More and more studies have demonstrated that chemotherapeutic agents can induce apoptosis in CRC by disrupting mitochondrial homeostasis. For example, Li and colleagues have proposed a novel inhibitor, RKI-1447, which disrupts mitochondrial homeostasis to enhance oxidative damage, inhibit cellular bioenergetics, and promote apoptosis in CRC cells by inducing ROS generation, mitochondrial depolarization, and mitochondrial fission. RKI-1447 may disrupt the ER-mitochondria crosstalk and MAMs integrity, leading to mitochondrial fragmentation.^162^ Another compound, sodium butyrate, a byproduct of anaerobic fermentation in the gastrointestinal tract, has been shown to have inhibitory effects on the growth of various cancers, including CRC. Sodium butyrate can induce cell cycle arrest and apoptosis through regulating DRP1 expression, thereby influencing mitochondrial fission and fusion.^163^ However, to shield tumors from the impacts of chemotherapy, cancer cells can swiftly eliminate damaged mitochondria through mitophagy.^164^ It has been observed that MFN2 is down-regulated in CRC. Subsequently, Sun et al found that the down-regulation of MFN2 expression by miR-17-5p impaired mitochondrial fusion, promoted mitochondrial fission, and stimulated mitophagy, ultimately leading to 5-fluorouracil resistance in CRC cells.^165^ These findings suggest that up-regulating MFN2 expression in CRC, thereby promoting mitochondrial fusion while inhibiting fission and mitophagy, may suppress CRC drug resistance and effectively prolong the survival of CRC patients.

On the other hand, some proteins localized to the MAMs contribute to CRC progression by influencing mitophagy. Among these proteins, PTEN-induced putative kinase 1 (PINK1) and Parkin play crucial roles in regulating mitophagy. PINK1 is a serine/threonine kinase protein that plays a key role in maintaining mitochondrial quantity and quality by participating in mitophagy. Accumulation of PINK1 is triggered by damaged OMM, leading to the recruitment of the E3 ubiquitin ligase Parkin to activate mitophagy in damaged mitochondria.^166^ The expression levels of PINK1/Parkin differ between CRC and normal tissues. Yin et al have revealed that PINK1 expression is down-regulated in human CRC tissues compared with normal tissues. Using two colitis-associated CRC mouse models, it was shown that a deficiency of PINK1 could increase the incidence of colon tumors.^167^ Conversely, overexpression of PINK1 may promote mitophagy by activating the p53 signaling pathway, reducing glycolysis, and boosting mitochondrial respiration.^167^ A recent study has reported that Parkin protein expression is lower in CRC tissues than in normal tissues. However, Parkin expression increases in late-stage CRC patients and serves as an independent predictor of improved survival rates.^168^

Several researchers have identified that targeting proteins involved in mitochondrial autophagy shows promise for the clinical management of CRC. Zhang et al discovered that aloe gel glucomannan induced colon cancer cell death through the PINK1/Parkin mitophagy pathway driven by mitochondrial damage, suggesting aloe gel glucomannan as a potential anticancer drug.^169^ Tanshinone IIA, isolated from the traditional Chinese medicine *Salvia miltiorrhiza*, is currently used in the treatment of various diseases, including cancer. It has been shown to promote CRC cell apoptosis in a mitochondria-dependent manner by inhibiting Parkin-mediated mitophagy.^170^ Cirsiliol is a natural product known to have anti-cancer effects on multiple tumors. Jiang et al found that cirsiliol inhibited colon cancer cell proliferation by reducing levels of mitophagy proteins (including PINK1, Parkin, and FUNDC1) and disrupting mitochondrial morphology.^171^ δ-valerobetaine, a novel dietary metabolite, exhibits cytotoxic effects in CRC cells through autophagy and apoptosis. Research by D’Onofrio et al indicates that δ-valerobetaine could enhance PINK1 and Parkin protein levels in the CRC SW-480 and SW-620 cells, supporting the activation of mitophagy as a contributing process in the mitochondria-related apoptosis.^172^ The non-coding RNA piR-823 is implicated in CRC development. Wang et al found that piR-823 interacted with PINK1, promoting its ubiquitination and proteasome-dependent degradation, thereby inhibiting mitophagy and cell apoptosis in CRC.^173^ These studies offer novel strategies for CRC treatment.

In conclusion, the inhibition of tumor cell proliferation in CRC treatment can be achieved through the modulation of mitochondrial fission/fusion or the regulation of mitochondrial autophagy. Meanwhile, consideration of gene expression, such as MFN2, which promotes drug resistance, is crucial.

## ER stress and CRC

### ER stress signaling at the ER-mitochondria interface

**Figure 5:**
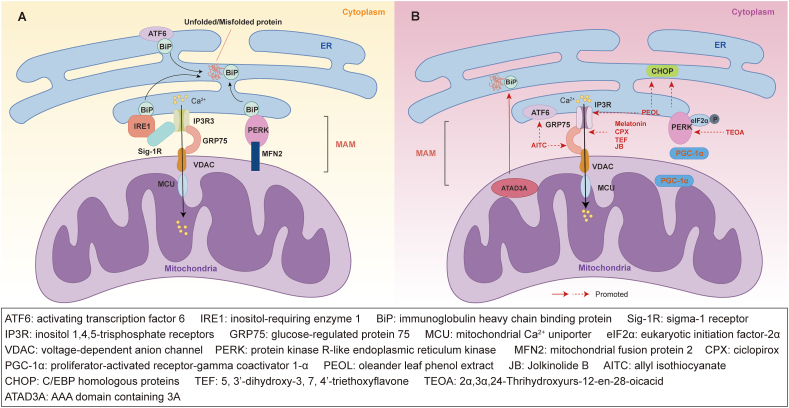
Endoplasmic reticulum (ER) stress and unfolded protein response (UPR) in ER, mitochondria, and mitochondria-associated ER membranes (MAMs). **(A)** ER stress and UPR in physiology. During ER stress, the competitive binding of unfolded or misfolded proteins to immunoglobulin heavy chain binding protein (BiP) displaces it from inositol-requiring enzyme 1 (IRE1), protein kinase R-like endoplasmic reticulum kinase (PERK), and activating transcription factor 6 (ATF6), leading to the activation of downstream signaling pathways. Mitochondrial fusion protein 2 (MFN2) directly interacts with PERK within the MAMs, acting as a tether. Under ER stress, IRE1 interacts with sigma-1 receptor (Sig-1R), promoting dimerization and facilitating Ca^2+^ transfer to the mitochondria through inositol 1,4,5-trisphosphate receptor (IP3R)-mediated mechanisms. **(B)** ER stress and therapy targets in colorectal cancer (CRC). ATPase family AAA domain-containing protein 3A (ATAD3A) stabilizes the folding of BiP to alleviate ER stress and promote cancer cell survival. Proliferator-activated receptor-gamma coactivator 1-α (PGC-1α) is highly expressed in 5-fluorouracil-resistant CRC cells and attenuates ER stress in 5-fluorouracil-resistant CRC cells, inhibiting cell apoptosis. Allyl isothiocyanate (AITC) induces ER stress by up-regulating ATF6, leading to cytosolic Ca^2+^ release, thereby modulating the apoptotic signaling pathway activated by the mitochondrial pathway. 2α,3α,24-Thrihydroxyurs-12-en-28-oicacid (TEOA) increases glucose-regulated protein 78 (GRP78) expression, promotes PERK and eukaryotic translation initiation factor-2α (eIF2α) phosphorylation, leading to up-regulation of C/EBP homologous protein (CHOP) expression. Oleander leaf phenol extract (PEOL) increases intracellular Ca^2+^ levels and CHOP expression in CRC cells, triggering ER stress-induced apoptosis. Jolkinolide B (JB), melatonin, ciclopirox (CPX), and 5, 3′-dihydroxy-3, 7, 4′-triethoxyflavone (TEF) influence Ca^2+^ homeostasis, leading to ER stress and cell death in CRC. The orange figure indicates overexpression. The blue figure indicates underexpression.

The homeostasis of the ER can be disrupted by various conditions, such as high protein demand, viral infections, expression of mutated proteins, hypoxia, energy deficiency, or excessive oxidative stress.^174^ These disruptions can impair the protein folding process, leading to the accumulation of unfolded or misfolded proteins and inducing ER stress. To cope with ER stress, eukaryotic cells activate the UPR, a complex signaling pathway that regulates the translation of specific proteins and the interaction with molecular chaperones related to protein folding and up-regulates signaling pathways responsible for targeting misfolded or unfolded proteins in the ER for ubiquitination and degradation, thereby helping cells adapt and restore ER homeostasis.^175^ UPR signaling is conveyed by at least three main stress sensors located on the ER membrane: inositol-requiring enzyme 1 (IRE1), PERK, and activating transcription factor 6 (ATF6).^176^ IRE1α is a transmembrane protein kinase/endoribonuclease that becomes activated and initiates its RNase activity when unfolded proteins accumulate in the ER, producing the active XBP1 transcription factor, which regulates genes associated with ER protein translocation, folding, and secretion, as well as the degradation of misfolded proteins.^177^ PERK is a kinase that phosphorylates the eukaryotic translation initiation factor-2α (eIF2α) during ER stress, leading to a temporary suppression of global protein synthesis.^178^ ATF6 moves from the ER to the Golgi apparatus, where it is cleaved by S1P and S2P, producing an active ATF6 fragment (ATF6p50), which migrates to the nucleus to activate the transcription of UPR target genes.^179^ Under normal conditions, the ER chaperone protein BiP binds to ER stress response proteins ATF6, IRE1, and PERK, inhibiting their activation and thus aiding in the proper folding of proteins in the ER. During ER stress, unfolded or misfolded proteins competitively bind to BiP, causing BiP to dissociate from IRE1, PERK, and ATF6, leading to the activation of the UPR signaling pathway.^176^^,^^180^ These pathways help to reduce protein translation during ER stress, thereby restoring the protein folding capacity of the ER (Fig. 5A).^175^^,^^181^

When ER stress is not resolved, the UPR can induce apoptosis.^182^ As stress persists, IRE1 activates apoptosis signal-regulating kinase 1 (ASK1) and c-Jun N-terminal kinase 1 (JNK1) through its kinase activity; eIF2α, phosphorylated by PERK, can activate the transcription factor ATF4, which further promotes the expression of the pro-apoptotic gene C/EBP homologous protein (CHOP), thus promoting cell apoptosis.^183^^,^^184^ Additionally, UPR activation can lead to the release of calcium ions from the ER, increasing mitochondrial Ca^2+^ uptake, triggering the opening of the mitochondrial permeability transition pore, leading to the release of cytochrome C and caspase activation; on the other hand, it can increase the expression of inflammatory factors, such as tumor necrosis factor α (TNFα), activating apoptotic pathways such as caspase-8. ER stress can increase the production of ROS, leading to oxidative damage and promoting cell apoptosis.^182^ Numerous studies have shown that UPR-induced apoptosis plays an important role in the treatment of various diseases, including cancer. In cancer therapy, UPR-induced apoptosis can serve as a therapeutic mechanism to kill cancer cells by activating apoptotic pathways, especially for those cancer cells that rely on high levels of UPR to maintain protein homeostasis.^185^ UPR can also increase the sensitivity of cancer cells to chemotherapy drugs, as the activation of UPR can increase oxidative stress and DNA damage in cancer cells, thereby promoting apoptosis.^186^

Furthermore, there exists a close interplay between the components of the MAMs and the UPR, with several ER chaperone proteins localized in the MAMs. Particularly, PACS2 and MFN2 are key proteins within MAMs, and their depletion can induce ER stress and UPR by uncoupling the structure of the ER from the mitochondria.^57^^,^^187^^,^^188^ MFN2 has been shown to directly interact with PERK within the MAMs, acting as a tether and playing a crucial role in the UPR to ER stress.^56^^,^^57^ Another MAMs tether protein, VAPB, has also been implicated in the UPR.^189^ Additionally, Kopp et al demonstrated that in response to ER stress, IRE1 interacted with the MAM-resident ER chaperone protein Sig-1R, promoting dimerization and facilitating Ca^2+^ transfer from the ER to the mitochondria through IP3R-mediated mechanisms.^190^ In addition to its pro-survival function, excessive ER stress and prolonged UPR activation can lead to cell death.^17^ For instance, in conditions of sustained ER stress, IRE1 can activate the spliced form of X-box binding protein 1 (XBP1s), triggering the synthesis of pro-apoptotic proteins and ultimately promoting apoptosis.^191^

### Roles of ER stress at the ER-mitochondria interface in CRC

Given that cancer arises and progresses within a challenging microenvironment, tumor cells often exploit the activation of the UPR as a mechanism for survival. Numerous studies have extensively investigated alterations in ER stress-related molecules in CRC using diverse genetic and pharmacological approaches. The MAMs play a crucial role in cancer proliferation and cell death, serving as a platform for regulating signaling events associated with ER homeostasis disruption. Targeting ER stress and UPR activation presents a novel therapeutic approach for intestinal tumors, including CRC (Fig. 5B).^192^, ^193^, ^194^, ^195^

ATAD3A, a nuclear-encoded mitochondrial protein involved in various mitochondrial functions, including dynamics and physiology, has been implicated in communication between the ER and mitochondria and linked to tumor metastasis.^196^, ^197^, ^198^ Clinical studies by Huang et al revealed a negative correlation between ATAD3A expression in tumor cells and the 5-year disease-free survival of CRC patients. ATAD3A has been shown to stabilize BiP protein folding, alleviate ER stress, and promote cancer cell survival, suggesting potential targeted therapies against ATAD3A for CRC treatment.^199^

Peroxisome proliferator-activated receptor-gamma coactivator 1-α (PGC-1α), highly expressed in mitochondria and MAMs, is pivotal in regulating mitochondrial biogenesis.^200^ Under hypoxic conditions, PGC-1α up-regulation reduces ROS production and has been associated with increased proliferation, enhanced motility, and spheroid formation in cancer cells.^201^ Yun et al have demonstrated elevated PGC-1α expression in 5-fluorouracil-resistant CRC cells, where it enhances mitochondrial biogenesis, oxidative phosphorylation, and antioxidant enzyme activity while inhibiting ROS production.^26^ Moreover, PGC-1α mitigates ER stress in 5-fluorouracil-resistant CRC cells, suppressing apoptosis. These findings highlight the significant role of PGC-1α in 5-fluorouracil-resistant CRC cells and propose it as a promising therapeutic target for CRC patients.^26^

Furthermore, a wide array of plant-derived compounds that modulate CRC progression by impacting ER stress offer promising new strategies for CRC treatment in the future. Zhang et al identified a triterpene, 2α,3α,24-Thrihydroxyurs-12-en-28-oicacid (TEOA), derived from kiwifruit, which induces autophagy in SW-620, leading to cancer cell death. Mechanistically, TEOA increases the expression of BiP in a dose-dependent manner and promotes the phosphorylation of PERK and phosphorylation of eIF2α, resulting in the up-regulation of downstream protein CHOP. These findings suggest the involvement of the PERK/eIF2α/CHOP pathway and ER stress in TEOA-induced autophagy in SW-620. Overall, this study demonstrates that TEOA induces autophagy in SW-620 through ER stress and mitochondrial autophagy, leading to cancer cell death.^202^ Another anti-tumor compound is oleander leaf phenol extract (PEOL), which can induce apoptosis in the HCT-116 xenograft mouse model. The mitochondrial pathway is implicated in PEOL-induced apoptosis, as evidenced by ROS production, reduction of mitochondrial membrane potential, and release of cytochrome C. Furthermore, PEOL elevates the intracellular Ca^2+^ levels in colon cancer cells, induces mitochondrial ROS production, and up-regulates CHOP expression, triggering ER stress-induced apoptosis. These results support the involvement of the mitochondrial pathway and ER stress in PEOL-induced apoptosis of colon cancer cells.^203^ Zhang and colleagues discovered that Jolkinolide B, isolated from the dried roots of the medicinal plant *Euphorbia fischeriana*, exhibits potent anti-tumor properties by inducing apoptosis in HT-29 and SW-620 cells. Further investigation has unveiled that Jolkinolide B induces ROS and ER stress, triggering ER Ca^2+^ release and mitochondrial Ca^2+^ uptake, leading to mitochondrial depolarization and cell death.^204^ Allyl isothiocyanate (AITC) is another biologically active phytochemical compound found in cruciferous vegetables, known for its chemopreventive and anti-cancer properties. Chiang et al reported that AITC inhibited proliferation, induced G2/M phase arrest, and promoted apoptosis in HT-29 cells. Specifically, AITC was shown to induce ER stress by up-regulating ER stress-associated proteins like ATF6 and elicit cytosolic Ca^2+^ release, thereby modulating the intrinsic apoptotic signaling pathway (mitochondrial pathway) in HT-29 cells.^205^ These findings suggest that TEOA, PEOL, Jolkinolide B, and AITC may serve as promising bioactive compounds for CRC treatment.

Moreover, beyond plant-derived compounds, studies have indicated that substances such as melatonin (an endogenous indoleamine hormone produced in the pineal gland), ciclopirox (an antifungal drug), and 5, 3′-dihydroxy-3, 7, 4′-triethoxyflavone (novel quercetin derivative), can affect the survival of CRC cells by modulating Ca^2+^ levels and inducing ROS generation, leading to ER stress. These compounds present novel therapeutic avenues for CRC treatment.^206^, ^207^, ^208^

## Discussion

In recent decades, there has been a growing interest in the intracellular communication between organelles. Our review primarily focuses on proteins involved in mitochondria-ER crosstalk in CRC (Table 1), as well as compounds targeting these proteins (Table 2). Moreover, the interaction sites between the ER and mitochondria, known as MAMs, are linked to various signaling pathways. MAMs play vital roles in regulating Ca^2+^ homeostasis, lipid metabolism, mitochondrial dynamics, and ER stress. Interestingly, alterations in the signaling pathways involving MAMs have been observed during the progression of cancers such as CRC, influencing cancer-related processes like proliferation, apoptosis, metastasis, and invasion. Therefore, understanding the role of MAMs in tumorigenesis is essential.

**Table 1:** The role of mitochondria-endoplasmic reticulum (ER) crosstalk-related proteins in colorectal cancer (CRC).

Mitochondria-ER crosstalk-related proteins	Localization	Effect	Reference
*Ca*^*2+*^*signaling*			
Sarco/endoplasmic reticulum Ca^2+^-ATPase (SERCA)	ER, mitochondria-associated endoplasmic reticulum membranes (MAMs)	Overexpression of SERCA2 stimulates the proliferation and migration of SW-480 cells by activating the mitogen-activated protein kinase (MAPK) and protein kinase B (AKT) signaling pathways.	97
		Decreased SERCA3 expression leads to progressive disturbances in cellular Ca^2+^ homeostasis.	98, 99
Inositol 1,4,5-trisphosphate receptors (IP3R)	ER, MAMs	IP3R3 facilitates Ca^2+^ signaling to mitochondria and exerts an anti-apoptotic effect in CRC cells.	106, 107, 108
Voltage-dependent anion channel (VDAC)	Mitochondria, MAMs	Activation of etoposide-induced protein 2.4 (EI24) mediated by p53 interacts with VDAC2, promoting the formation of ER-mitochondria contacts, enhancing Ca^2+^ flux from the ER to the mitochondria, and promoting DNA damage-induced apoptosis in HCT116 cells.	110
		VDAC1 is overexpressed in CRC, and its down-regulation can hinder the proliferation of CRC cell lines.	111, 112
Mitochondrial Ca^2+^ uniporter (MCU)	Mitochondria, MAMs	Up-regulation of MCU enhances mitochondrial Ca^2+^ uptake, promoting mitochondrial biogenesis and accelerating CRC development.	116
*Lipid metabolism*			
Ankyrin repeat domain-containing protein 22 (ANKRD22)	Mitochondria, ER, cytoplasm	ANKRD22 promotes glycolysis and collaborates with the lipid transfer protein extended synaptotagmin-1 (E-Syt1) to stimulate lipid transport to the mitochondria, driving metabolic reprogramming in CRC cells.	136
Tp53-regulated inhibitor of apoptosis 1 (TRIAP1)	Mitochondria, cytoplasm, MAMs	The expression of TRIAP1 supports cancer cell proliferation and tumorigenesis in CRC cell models. Depletion of TRIAP1 induces external perturbations in mitochondria, leading to ER-mediated alterations in lipid homeostasis and triggering a p53-mediated stress response.	28
Long chain acyl CoA synthetase 4 (ACSL4)	Mitochondria, ER, MAMs	ACSL4 is up-regulated in CRC tissues and associated with a poor prognosis in CRC patients.	125, 139, 215
*Mitochondrial dynamics*			
Dynamin-related protein 1 (DRP1)	Mitochondria, cytoplasm, MAMs	DRP1 deficiency primarily inhibits cell proliferation and enhances apoptosis in human CRC cells by increasing cytochrome C release and reducing mitochondrial membrane potential.	160
Mitochondrial fusion protein 2 (MFN2)	Mitochondria, ER, MAMs	Down-regulation of MFN2 signifies a poor prognosis and acts as an independent risk factor for CRC prognosis.	161
		The down-regulation of MFN2 expression by miR-17-5p impairs mitochondrial fusion, promotes mitochondrial fission, and stimulates mitophagy, ultimately leading to 5-fluorouracil resistance in CRC cells.	165
PTEN induced putative kinase 1 (PINK1)/Parkin	Mitochondria, ER, cytoplasm, MAMs	A deficiency of PINK1 can increase the incidence of colon tumors.	167
		The non-coding RNA piR-823 interacts with PINK1, promoting its ubiquitination and proteasome-dependent degradation, thereby inhibiting mitophagy.	173
		Parkin expression increases in late-stage CRC patients and serves as an independent predictor of improved survival rates.	168
*ER stress*			
AAA domain-containing protein 3A (ATAD3A)	Mitochondria, cytoplasm, MAMs	ATAD3A stabilizes the folding of immunoglobulin heavy chain binding protein (BiP), alleviates ER stress, and promotes cancer cell survival. The expression of ATAD3A in tumor cells is negatively correlated with the 5-year disease-free survival of CRC patients.	199
Proliferator-activated receptor-gamma coactivator 1-α (PGC-1α)	Mitochondria, cytoplasm,	Elevated PGC-1α expression in 5-fluorouracil-resistant CRC cells enhances mitochondrial biogenesis, oxidative phosphorylation, and antioxidant enzyme activity while inhibiting reactive oxygen species production. PGC-1α mitigates ER stress in 5-fluorouracil-resistant CRC cells, suppressing apoptosis.	26

**Table 2:** Compounds regulate mitochondria-endoplasmic reticulum (ER) crosstalk in colorectal cancer (CRC).

Compounds	Mechanism	Reference
*Ca*^*2+*^*signaling*		
Resveratrol	Resveratrol and its derivative paclitaxel inhibit ATP synthase, subsequently diminishing sarco/endoplasmic reticulum Ca^2+^-ATPase (SERCA) activity, resulting in cancer cell death.	100
Dihydroartemisinin	It induces ER stress by inhibiting SERCA activity, leading to Ca^2+^ overload and apoptosis in HCT-116 cells.	102
α-hederin	It induces paraptosis by increasing the expression of inositol 1,4,5-trisphosphate receptor (IP3R) and activating the Ca^2+^ signaling pathway in CRC. It induces paraptosis in 5-fluorouracil-resistant CRC cells and reduces the growth of 5-fluorouracil-resistant CRC xenografts.	105
Remimazolam	Targeting translocator protein (TSPO) with remimazolam inhibits the TSPO/voltage-dependent anion channel (VDAC) pathway, resulting in increased apoptosis in CRC cells.	109
Erastin	It binds to VDAC, leading to the opening of the mitochondrial permeability transition pore and caspase-9-dependent cell apoptosis in HT-29 cells.	113, 216, 217
Sulindac sulfone	It induces apoptosis in human CRC cells by binding to VDAC1/2.	114, 115
*Lipid metabolism*		
Emodin	It binds directly to the long-chain acyl CoA synthetase 4 (ACSL4) targets, inhibiting ACSL4 in HCT-116 cells and reducing cell proliferation and invasion in *in vitro* experiments.	27
*Mitochondrial dynamics*		
RKI-1447	It disrupts the ER-mitochondria crosstalk and mitochondria-associated ER membrane integrity, leading to mitochondrial fragmentation and apoptosis in CRC cells.	162
Sodium butyrate	It can induce cell cycle arrest and apoptosis through regulating dynamin-related protein 1 (DRP1) expression in CRC cells.	163
Aloe gel glucomannan	It induces colon cancer cell death through the PTEN-induced putative kinase 1 (PINK1)/Parkin mitophagy pathway driven by mitochondrial damage.	169
Tanshinone IIA	It promotes CRC cell apoptosis in a mitochondria-dependent manner by inhibiting Parkin-mediated mitophagy.	170
Cirsiliol	It inhibits colon cancer cell proliferation by reducing levels of mitophagy proteins (including PINK1, Parkin, and FUN14 domain containing 1/FUNDC1) and disrupting mitochondrial morphology.	171
δ-valerobetaine	It enhanced PINK1 and Parkin protein levels in SW-480 and SW-620 cells, supporting the activation of mitophagy as a contributing process in the mitochondria-related apoptosis.	172
2α,3α,24-Thrihydroxyurs-12-en-28-oicacid	It increases the expression of immunoglobulin heavy chain binding protein (BiP) in a dose-dependent manner and promotes the phosphorylation of protein kinase R-like endoplasmic reticulum kinase (PERK) and eukaryotic translation initiation factor-2α (eIF2α), resulting in the up-regulation of downstream protein C/EBP homologous protein (CHOP) and autophagy in SW-620 cells.	202
*ER stress*		
Oleander leaf phenol extract	It elevates the intracellular Ca^2+^ levels in colon cancer cells, induces mitochondrial reactive oxygen species production, and up-regulates CHOP expression, triggering ER stress-induced apoptosis in the HCT-116 xenograft mouse model.	203
Jolkinolide B	It induces reactive oxygen species and ER stress, triggering ER Ca^2+^ release and mitochondrial Ca^2+^ uptake, leading to mitochondrial depolarization and apoptosis in HT-29 and SW-620 cells.	204
Allyl isothiocyanate	It induces ER stress by up-regulating ER stress-associated proteins like activating transcription factor 6 (ATF6) and eliciting cytosolic Ca^2+^ release, thereby modulating the intrinsic apoptotic signaling pathway (mitochondrial pathway) in HT-29 cells.	205

While previous studies have explored the potential of targeting MAMs for cancer therapy, most have primarily focused on the Ca^2+^ signaling-mediated pathways leading to cell apoptosis.^24^ As the involvement of MAMs in autophagy and inflammatory signaling is being validated, further research is warranted to elucidate the mechanisms through which MAMs regulate cancer cell death. Some studies have demonstrated crosstalk between different signaling pathways at MAMs. For instance, IP3R, a crucial Ca^2+^ channel protein, not only maintains Ca^2+^ homeostasis between mitochondria and the ER but also interacts with mitophagy-related proteins to influence organelle turnover.^209^ Another MAM-resident protein, PGC-1α, impacts mitochondrial biogenesis in CRC cells and attenuates ER stress to inhibit apoptosis.^26^ Therefore, considering the interplay of diverse signaling pathways is essential when exploring therapeutic strategies for CRC.

Furthermore, the lack of specific markers for MAMs poses challenges in accurately assessing cellular substructures.^210^ Therefore, investigating the functions of MAMs in cancer cells while sparing normal cells presents a significant challenge. In recent years, numerous attempts have been made to address this issue. For example, engineered based on the human NAF-1/CISD2 protein, NAF-1^44−6^ is a peptide that selectively penetrates human epithelial breast cancer cells instead of normal cells, targeting and eliminating mitochondria and the ER to induce apoptosis, ferroptosis, and necroptosis.^211^ Concurrently, a prodrug named lysergic acid amide was identified for its ability to modulate tumor cell mitochondrial-ER contacts, enhancing ferroptosis while exhibiting minimal effects on normal cells, thus highlighting its biological safety.^212^ Furthermore, Gao et al, through a series of animal experiments, found that increasing MFN2 in CD8^+^ T cells could strengthen the mitochondria-ER interaction, which enhances the efficacy of cancer immunotherapy without direct intervention in cancer cells, minimizing side effects in normal cells.^213^ Additionally, the development of THTTPy-PTSA, a light-activated probe, has shown promise in sequentially targeting the ER and mitochondria. It can amplify the ER stress response by modulating the ER-mitochondria network cascade and promote the release of tumor-associated antigens and damage-associated molecular patterns, leading to immunogenic cell death. Notably, a cancer vaccine based on THTTPy-PTSA has demonstrated low toxicity and good biosafety *in vivo*, underscoring the potential of these strategies in advancing cancer therapeutics.^214^ These studies collectively indicate that it is possible to develop cancer therapeutic strategies that can precisely target the MAMs in cancer cells while minimizing harm to healthy cells.

In summary, MAMs serve as crucial biochemical and physical contact points that bridge the ER and mitochondria. Proteins such as ACAT1, ACSL4, DRP1, GRP75, HK2, IP3R, MFN1/2, and PINK1 are among the MAMs proteins involved in maintaining the structural integrity and functional regulation of MAMs. Further research is anticipated to unveil additional proteins constituting MAMs structures and elucidate the intricate interactions among these diverse MAMs proteins. Dysregulation or structural changes in certain MAMs proteins have been linked to alterations in organelle functions, contributing to tumorigenesis. These MAMs proteins represent promising targets for drug interventions and cancer therapies. Additionally, the discovery of cancer-specific MAM-resident proteins may open new avenues for cancer treatment. Given the growing evidence supporting the crucial role of MAMs in CRC progression, comprehensive investigations into the structural, functional, and dynamic changes of MAMs proteins are crucial for tailored precision therapies in CRC. Notably, research on MAMs proteins in CRC lags behind that in other tumor types, underscoring the urgent need for further studies to elucidate and validate the roles and molecular mechanisms of MAMs proteins in CRC. Such endeavors could offer fresh insights for the treatment of CRC and other related diseases.

## CRediT authorship contribution statement

**Lanshu Xiao:** Data curation, Formal analysis. **Yao Wei:** Formal analysis, Data curation, Writing – original draft. **Yiping Qin:** Conceptualization, Project administration, Writing – review & editing. **Bianqin Guo:** Writing – review & editing, Conceptualization.

## Funding

This work was supported by the Chongqing Natural Science Foundation (China) (No. CSTB2023NSCQ-MSX0845, CSTB2024NSCQ-MSX0591), the Shapingba District 2024 Science and Health Joint Medical Research Project, Chongqing, China (No. 2024SQKWLHMS009), the Open Research Fund Program of the NHC Key Laboratory of Personalized Diagnosis and Treatment for Nasopharyngeal Carcinoma (China) (No. 2023NPCCJ01), and Open Research Fund Program of the Key Laboratory of Molecular Biology for Infectious Diseases of 10.13039/501100004374Chongqing Medical University, China (No. 202406).

## Conflict of interests

The authors declared no conflict of interests.
